# Controllable Cell Deformation Using Acoustic Streaming for Membrane Permeability Modulation

**DOI:** 10.1002/advs.202002489

**Published:** 2020-12-21

**Authors:** Xinyi Guo, Mengjie Sun, Yang Yang, Huihui Xu, Ji Liu, Shan He, Yanyan Wang, Linyan Xu, Wei Pang, Xuexin Duan

**Affiliations:** ^1^ State Key Laboratory of Precision Measuring Technology & Instruments Tianjin University Tianjin 300072 China; ^2^ College of Precision Instrument and Opto‐electronics Engineering Tianjin University Tianjin 300072 China

**Keywords:** acoustic streaming, cell deformation, drug delivery, hydrodynamic force, membrane permeability

## Abstract

Hydrodynamic force loading platforms for controllable cell mechanical deformation play an essential role in modern cell technologies. Current systems require assistance from specific microstructures thus limiting the controllability and flexibility in cell shape modulation, and studies on real‐time 3D cell morphology analysis are still absent. This article presents a novel platform based on acoustic streaming generated from a gigahertz device for cell shape control and real‐time cell deformation analysis. Details in cell deformation and the restoration process are thoroughly studied on the platform, and cell behavior control at the microscale is successfully achieved by tuning the treating time, intensity, and wave form of the streaming. The application of this platform in cell membrane permeability modulation and analysis is also exploited. Based on the membrane reorganization during cell deformation, the effects of deformation extent and deformation patterns on membrane permeability to micro‐ and macromolecules are revealed. This technology has shown its unique superiorities in cell mechanical manipulation such as high flexibility, high accuracy, and pure fluid force operation, indicating its promising prospect as a reliable tool for cell property study and drug therapy development.

## Graphical abstract

This article presents a novel hydrodynamic force loading platform based on acoustic streaming generated from a gigahertz ultrasonic device for controllable cell deformation and real‐time 3D cell shape analysis. The platform is applied for cell membrane permeability modulation, and a novel phenomenon that fast and periodic cell deformation will facilitate the intracellular transportation of large cargos is observed.

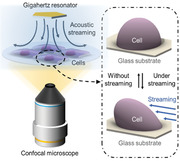

## 1 Introduction

Living cells are always undergoing continuous internal and external mechanical forces. Response to these forces can trigger the deformation of cells, which will further influence the functions and biological properties of cells.^[^ ^1^, ^2^, ^3^, ^4^ ^]^ Exogenous mechanical stimulation can regulate the growth,^[^ ^5^ ^]^ differentiation,^[^ ^6^ ^]^ intercellular signaling of cells,^[^ ^7^ ^]^ providing important guidance for tissue engineering and regenerative medicine.^[^ ^8^, ^9^ ^]^ Besides, controllable force exertion and cell extrusion are demonstrated to have profound effects on transmembrane transportation by producing transient disruptions on the membrane,^[^ ^10^, ^11^ ^]^ which can be used for drug targeting^[^ ^12^ ^]^ and gene engineering.^[^ ^13^ ^]^ However, details on the mechanism of cell deformation process and the relationship between deformation and membrane permeability are still less studied. Thus, exploiting microscaled force manipulation systems, which enables mechanical operations at cellular level with high controllability and real‐time analysis become essential.

Recent advances in micro‐ and nanotechnology have extended the research tools for cell force loading and mechanical deformation studies. These tools can be generally classified into contact and noncontact approaches, according to their way of applying forces to cells. A majority of these approaches, such as micropipette aspiration,^[^ ^14^, ^15^ ^]^ atomic force microscopy (AFM),^[^ ^16^ ^]^ microchannel cell squeezing,^[^ ^17^, ^18^ ^]^ magnetic manipulators,^[^ ^19^, ^20^ ^]^ and surface microstructures^[^ ^5^, ^8^, ^21^, ^22^ ^]^ require direct contact between cell membrane and external apparatus. Each of these techniques has its own strengths and its most appropriate applications. However, due to the direct contact in these methods, contamination of the surfaces and irreversible damages to cells are typical issues. Noncontact approaches such as optical stretchers,^[^ ^23^, ^24^ ^]^ acoustic tweezers^[^ ^25^ ^]^ and dielectrophoresis (DEP)^[^ ^26^, ^27^ ^]^ taking advantage of field forces make a step forward in controlling cell deformation. Among these approaches, hydrodynamic method, using the flow force generated from bubble oscillation^[^ ^28^, ^29^ ^]^ or shear and extensional flow,^[^ ^30^, ^31^, ^32^, ^33^ ^]^ has been successfully applied in cell deformation studies. Nevertheless, the assistance of bubbles or specific microfluidic structures is required in these hydrodynamic systems. Bubbles may increase the difficulty to achieve highly accurate control due to the uncertainty in bubble generation and positioning, and specific microfluidic structures in cells’ culturing environments will limit the flexibility in tuning the force pattern and the in situ cell culture and analysis. Besides, cell shapes analyzed from micrographs or 2D slices limit the perceptions of real cell morphology and the possibility for multidirectional analysis. Thus, a 3D real‐time mechanical deformation system which can achieve precise control with high flexibility, and have no restrictions on exogenous materials and operating environments, is still high in demand.

Here, we present a noncontact platform for the controllable and tunable cell deformation based on the hydrodynamic force generated from acoustic streaming under gigahertz (GHz) resonator excitation. Previous studies from our group have revealed the ability of such microfabricated resonator to generate localized and high‐speed fluid motion due to the rapid attenuation of acoustic energy in liquid environment, and its biomedical applications for microscale fluidic mixing,^[^ ^34^ ^]^ tuning biomolecule–surface interactions^[^ ^35^, ^36^ ^]^ and particle manipulations.^[^ ^37^ ^]^ We have reported a drug delivery method based on gigahertz resonator,^[^ ^38^ ^]^ in which the delivery mechanism and the acoustic streaming generation were not fully explored. Following studies have tried to model the cell response to gigahertz acoustic field using unilamellar vesicles,^[^ ^39^ ^]^ in which material exchange over the synthetic membrane was observed. In this article, we integrated the miniaturized acoustic resonator with the confocal microscope for the real time 3D analysis of the cell mechanical responses under acoustic stimulation. The platform can realize gradual and restorable cell deformation with tunable deformation extent by power adjustment. We also realized reciprocating and pulsating cell shape change under square‐waved fluid excitation. To our knowledge, this is the first real‐time 3D observation of tunable cell deformation under complex hydrodynamic force patterns. The application of this system in modulating cell membrane permeability were further studied. Reorganization of the cell membrane during deformation process was observed, and relationship between the cell deformation character and the cell membrane permeability was thoroughly studied. A novel phenomenon is revealed by our system that fast and periodic cell deformation under hydrodynamic stimulation will facilitate the membrane permeability for intracellular transportation applications. Comparing with acoustics at lower frequencies, this platform has shown its particular advantage on providing large body force for fluid driven due to the fast‐attenuation of gigahertz vibration, which facilitates the generation of localized high‐speed stream flow at approximately m s^−1^ level and enables the microscale force control over a wide range of several tens of micro Newton. No assistance from external structures, such as sharp edges, needles, or bubbles is needed to couple with a transducer for acoustic energy transfer and fluid flow magnification,^[^ ^40^, ^41^, ^42^ ^]^ thus increases the system integration and stability and decreases the possibility to induce cell damage due to the sharp structures. The effective range can be adjusted by tuning the distance or resonator size which enables both localized cell manipulation or large‐scaled cell batch processing. There are also no limitations on specific cell growth environments, which perfectly fits the subsequent in situ cell cultivation and conventional cell detection techniques. In summary, this platform is a promising tool for the development of new cell manipulation and cell therapeutic methods.

## 2 Results and Discussion

### 2.1 Simulation and Force Characterization of the Acoustic Streaming under GHz Device Excitation

**Figure 1:**
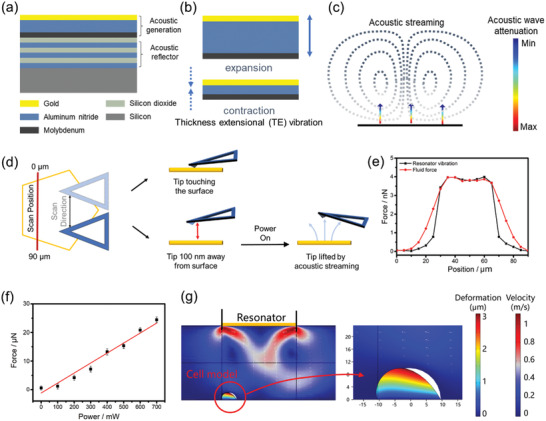
Acoustic streaming characterization and cell behavior simulation. a) Vertical structure of the gigahertz resonator. b) Resonator vibration. The resonator works at thickness extensional (TE) mode in which longitudinal expansion and contraction occurs to generate vertically propagated acoustic wave. c) The acoustic wave transmits into liquid, and acoustic streaming is generated due to the acoustic attenuation. d) Schematic of the AFM force detection of the acoustic streaming. The yellow pentagon indicates the resonator, and the red line shows the detected position. e) Detected force distribution of the resonator vibration (black) and the fluid motion (red) under 0.1 mW power supply. f) Relationship between the power applied to the resonator and the fluid force measured by the ultrasensitive force sensor (*n* = 3). g) Simulation of cell deformation under acoustic streaming stimulation. The cell is represented by an elastic semicircle surrounded by water and is placed 100 µm away from the resonator surface. White arrows indicate flow directions, and color bars indicate streaming velocity distribution and cell deformation extent.

The working mechanism of gigahertz resonator and acoustic streaming generation is illustrated in **Figure** **1**a–c. The resonator is mainly composed of a piezoelectric layer (aluminum nitride, AlN) sandwiched between two electrodes. When applying an external vertical electric field through the electrode, geometric deformation is triggered within the piezoelectric material due to the inverse piezoelectric effect, thus generates mechanical vibration. The resonator was designed working at thickness extensional (TE) mode, in which symmetric displacement occurs on the upper and lower boundary of the piezoelectric layer to generate vertically propagating longitudinal wave. The resonant frequency is mainly governed by the thickness of the piezoelectric layer and can be estimated by the following equation:

(1) $$f=\frac{1}{2d}\sqrt{\frac{c}{\rho }}$$

where *d* is the thickness of the piezoelectric material, *c* and *ρ* are the material stiffness coefficient and density respectively.^[^ ^43^ ^]^ The thickness of the AlN thin film can be precisely controlled from nanometers to micrometers during deposition process, thus enables the device to achieve an ultrahigh vibration frequency over gigahertz. The working area of the device was pattern into a pentagon shape to minimize unwanted lateral‐wave resonance and achieve higher energy utilization.

When the resonator works in liquid, acoustic wave propagates into liquid and attenuation occurs due to the fluid viscosity.^[^ ^44^ ^]^ The acoustic propagation can be described as follow:

(2) $$v=v_{0}\;e^{-\beta z}$$

where *v*_0_ is the initial velocity amplitude, and z is the direction of propagation. *β* is the attenuation coefficient which is defined by:

(3) $$\beta =\frac{\omega^{2}}{2\rho {c_{l}}^{3}}\left(\frac{4}{3}\mu +\mu_{B}\right)$$

In which *ω* is the angular frequency, *c*_l_ is the sound speed in liquid, *ρ*, *μ*, and *μ*_B_ denote the density, dynamic viscosity and bulk viscosity of the liquid. The equation indicates that the acoustic attenuation coefficient is proportional to *ω* ^2^, and acoustic wave of a higher frequency will attenuate much more rapidly. The decay length of an acoustic wave is defined by the distance where the amplitude decreased to 1/*e* of its original state, which is 1/ *β*. We can calculate that the decay length of a 1.64GHz acoustic wave is only 17.4 µm in water. In this paper, a minimum distance of 100 µm was used for cell manipulation where the acoustic wave already attenuated to 0.3% of its initial amplitude. Therefore, the influence of the acoustic pressure on cells can be ignored in our study.

The acoustic energy dissipation into liquid will cause a body force (*F*) for fluid driven, which is expressed as follow:

(4) $$F\;=\;2\beta \rho {v_{0}}^{2}e^{-2\beta z}$$

where *v*_0_ is the initial velocity amplitude. In conclusion, acoustic waves with higher frequencies have a shorter travelling distance, meanwhile generate a larger local force for liquid actuation. For example, compared with acoustic wave of 1.5 MHz frequency, the initial body force generated by a 1.5 GHz wave at the solid–liquid interface can be 10^6^ larger, indicating this gigahertz resonator to be a perfect tool for generating micro‐scaled high‐speed fluid motion. Besides frequency, the resonator size also influences the generated acoustic streaming. A simulation study is given in Figure S1 in the Supporting Information for detailed discussion.

To understand the cell deformation under such acoustic streaming, we first characterized the force distribution in such small‐scale liquid flow with atomic force microscope (AFM) and ultrasensitive force sensor, followed by a simulation of the cell behavior under the hydrodynamic force field. Schematic and the result for AFM force mapping of the acoustic steaming is given in Figure 1d,e. AFM tip was scanning along a 90 µm line above the resonator. Before fluid force measurement, resonator vibration was detected to confirm the relative position of the AFM tip with the active resonant region. The up‐lifting fluid motion above the resonant area under 0.1 mW power supply was tested by placing the AFM tip 100 nm away from the solid surface. When the tip was located outside the pentagon resonant region, the detected force almost equals to zero. By gradually sweeping the tip toward the resonator active area, fluid force increased sharply. In the pentagon center, the force shows a relatively flat distribution, and a vertical force of about 4 nN was recorded. The distribution of the fluid force is well matched with the shape of the device, which confirms the generation of the localized fluid motion based on gigahertz nanoscale oscillation. The AFM results provide a clear observation of the fluid force distribution, however due to the limits of the AFM system it cannot precisely measure larger forces. To further characterize the streaming at larger acoustic energy, we then detected the streaming force under different applied power using a commercialized miniaturized force sensor (Aurora Scientific, 405A). The result in Figure 1f indicates that the strength of the force is linearly proportional to the power applied from a few to a few tens of µN, demonstrating a good controllability of this force loading platform over a wide adjustable force range. This is the first detailed force characterization on the acoustic streaming generated from gigahertz ultrasonic device. It provides a new perspective and a deeper insight for the mechanism studies of the interactions between acoustic streaming and biomaterials. We also provided a streaming velocity study by recording the microparticle movement in liquid, and the video and data analysis are given in Figure S2 and Video S1 in the Supporting Information.

To further understand the effects of the streaming force on the cell deformation, simulation of the interaction between the acoustic streaming and a single cell is carried out. As shown in Figure 1g, a cell model was placed 100 µm away from the GHz resonator. When the device works in liquid environment, acoustic streaming is generated and hydrodynamic force is applied along the cell surface. Under such flow field excitation, the cell is squeezed from the semicircular (white area) to a compressed condition. The most significant displacement occurs at the top of the cell, while the bottom of the cell remains at its original status. This displacement difference results in the oblique deformation of the cell. The dynamic simulation result is given in Video S2 (Supporting Information), from which the transient response of the cell under acoustic streaming can be obtained. To explore the influence of the horizontal relative position between the cell and the resonator on cell deformation, simulation results under different cell locations were compared as well (Figure S3, Supporting Information), and obvious cell deformation can be obtained when cells are located within 100 µm from the center of the resonator. Since a 2D simulation cannot fully represent the real 3D space, we also performed a 3D simulation under the same condition as Figure 1g (Figure S4 in Supporting Information). Comparing the results of 2D and 3D simulations, similar cell deformation feature and deformation extent can be seen. Thus, in the following analysis, 2D simulation will be used for modeling simplification.

### 2.2 Controllable and Restorable Cell Deformation

**Figure 2:**
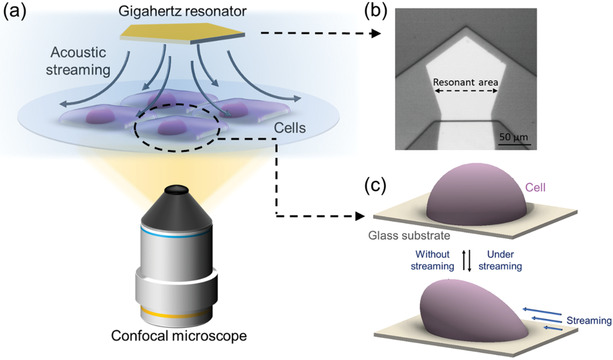
Controllable cell deformation platform. a) Schematic of the controllable cell deformation and real‐time 3D observation system based on the acoustic streaming generated from gigahertz ultrasonic device. b) Optical image of the gigahertz resonator. c) Cell deformation under the stimulation of the acoustic streaming.

After simulation of the cell behaviors under the acoustic streaming, we experimentally recorded the real‐time cell deformation process by integrating the GHz resonator with a confocal microscope to provide a 3D dynamic characterization of the cell. The schematic of the GHz hydrodynamic cell deformation and 3D cell morphology recording system is shown in **Figure** **2**. A bulk acoustic resonator with frequency of 1.64 GHz was utilized to excite acoustic streaming. The resonator was inserted into the solution with its resonant area facing down toward the cells seeded Patri dish, and the vertical distance was controlled to 100 µm according to our simulation in Figure 1g. Under this condition, the size of the major streaming vortex area (Figure S3, Supporting Information) is around 150 µm in diameter, and will only deform the cells near and within the resonator working area. Thus, this distance was used to maximize force utilization for localized cell deformation. Z‐stacking function in confocal microscope was applied to obtain the 3D cell morphology (Video S3 and Figure S5, Supporting Information). By continuously recording the complete volumes of the cells, real‐time analysis of the cells before, during and after the streaming stimulation was achieved.

**Figure 3:**
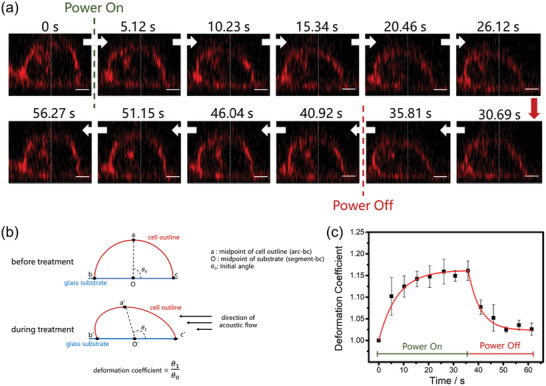
Cell deformation process and cell shape characterization. a) Real‐time cell outline deformation before, during and after the treatment of acoustic streaming (400 mW applied). The cell is squeezed to the left by the acoustic streaming, and gradually restored to its original shape after stopping the stimuli. White thin dashed line indicates the original cell central position. Scale bar is 5 µm. b) Calculation method of cell deformation coefficient. c) Cell deformation coefficient change with the treating time (*n* = 3).

A typical recorded cell deformation dynamic process is given in Video S4 (Supporting Information). The video provides an intuitive understanding of the cell deformation in the 3D space, and enables the cell shape characterization from different directions. To maximize the effect of the acoustic streaming, here we extract the vertical sections of the treated cell along the maximum cell deformation direction for the following analysis, as shown in **Figure** **3**a. At the initial stage, the cell maintains original hemispherical shape. When applying a power of 400 mW to the resonator, the stimulated acoustic flow flushes the substrate and forms a lateral streaming from right upon the cell. Since the bottom of the cell adheres to the substrate firmly, the fluid force pushes the cell deflecting to the left. Such deformation is happened in a very short time (≈5 s). Due to the press, the right half of the cell is compressed, meanwhile the left half is ballooned, which agrees with the aforementioned simulation results. With the increase of the treating time, the deflection angle gets larger and remains almost stable after 15 s. In order to analyze the long‐term influence of the streaming on the cell, we also recorded the cell shape change after stopping the stimulation. A quick recovery from the cell stretch status happens within 5 s after turning off the power, and the cell shape continues to change and recover to its original condition after 20 s relaxation.

To quantify the deformation feature of cells, we introduce an indicator based on the angle of cell deflection, as shown in Figure 3b. Longitudinal section of a cell can be simplified as an arc (arc‐bc) with its two end points (point b and c) on the substrate. We define the mid of the cell outline to be the point (point a) which divide the arc into two parts with the same length (arc‐ab = arc‐ac), and the mid of the substrate (point O) to be the center of the area on the petri dish occupied by the cell (segment bc), that angle *θ* (∠aOc) represents the cell deflection. When cell is pressed by the streaming flow, midpoint of cell outline moves along the direction of acoustic streaming due to the cell deflection, thus making angle *θ* increases (from *θ*_0_ to *θ*_1_). The deflection coefficient (DC) is defined by the ratio of these two angles:

(5) $$\mathrm{Deflection}\;\mathrm{coefficient}\left(\mathrm{DC}\right)=\frac{\theta_{1}}{\theta_{0}}$$

which can be used to quantify the cell deformation. DC should be equal to or larger than 1 ( = 1 indicates cell original shape), and a larger DC indicates a more severe deformation.

The cell DC is extracted and plotted with different time in Figure 3c. The coefficient gradually increased to about 1.15 when maximum cell deformation occurs at 35 s. The most rapid shape change happens within 5 s after the power is applied, and then the changing rate slowed down until the cell shape reaches the equilibrium position. When the stimulation is stopped, a quick decrease of the coefficient is also happened in the first 5 s, which is consistent with the observation from Figure 3a. The DC keeps decreasing in the following recordings and the final value reaches to almost 1, indicating that the streaming force under this power intensity has negligible long‐term influence on the integrity of the cell and the shape can finally recover to its original state. The results also confirm that the cell deformation extent produced by our platform is time controllable.

**Figure 4:**
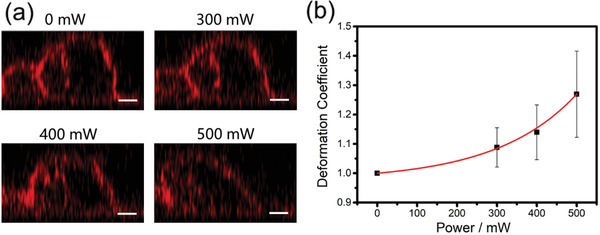
Cell deformation under different powers. a) The cell was treated with a time period of 5 s. Scale bar is 5 µm. b) Relationship between deformation coefficient and the power applied (*n* = 3).

To further investigate the influence of applied power on the cell deformation, different powers were applied to the same cell, and the cell deformations after treated for the same periods are given in **Figure** **4**a (also see Video S5 in the Supporting Information). It is observed that the deformation extent increased by increasing the power intensity. When applying 300 mW power, the cell shows slight deflection. When the power is increased to 500 mW, the cell is severely compressed to an oblate shape, and the deformation coefficient increased to more than 1.25 (Figure 4b). The result reveals that the cell deformation extent can be delicately controlled in our platform by tuning time duration or power of the stimulation, proving it to be a powerful tool for cell shape manipulation. Besides cell deformation, we also discussed the influence of fluid on cell detachment. We have found that cell peeling may occur when treating a separated single cell of a small attaching area on the substrate (Figure S6 and Video S6 in the Supporting Information) or using larger acoustic powers (larger than 500 mW), and a detailed study is given in S7 (Supporting Information).

### 2.3 Complex Cell Shape Manipulation under Periodic Streaming Excitation

**Figure 5:**
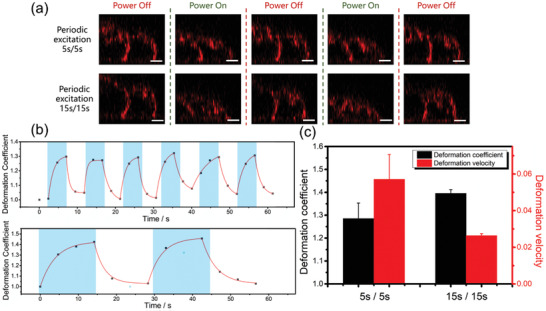
Cyclic cell deformation under periodic streaming excitations. a) Cell shape change in two cycles under 5 s/5 s excitation and 15 s/15 s excitation. Scale bar is 5 µm. b) Deformation coefficient variation with time under two excitation methods. Blue area indicates the period when the power is applied. c) Comparation of cell deformation extent and average deformation velocity under different excitation methods (*n* = 3).

The previous data has proved that the cell deformation or the recovery process closely follows the period of the acoustic streaming. Thus, we assume that if applying periodically acoustic streaming with tunable duration, the treated cells would be excited by cyclic hydrodynamic force, and exhibiting an oscillating shape change. To verify our analysis, we performed cell deformation experiments using different periodic excitation methods, as shown in **Figure** **5**. The period of the acoustic streaming is controlled by applying and tuning a square‐waved power to the GHz resonator. Two kinds of pulse durations, 5 s/5 s (indicating a square wave with 5 s power on and 5 s power off in a period of 10 s) and 15 s/15 s were used. The real‐time measurement of the streaming force under these two pulse excitations is given in Figure S8 (Supporting Information). The force result shows the same variation as the applied electric signal, indicating the acoustic streaming can be well controlled under periodic gigahertz excitation. Video S8 (Supporting Information) shows the cell status under these two stimulation methods, the maximum and minimum deformation within two cycles are plotted in Figure 5a. Repeating cell extrusion and recovery process are observed, which are closely following the variation of the applied power. Extracted cell deformation coefficient (DC) is given in Figure 5b, from which we can see that the feature and the extent of the cell deformation in each cycle is consistent, indicating a good repeatability. To describe the feature of cell deformation under cyclic acoustic streaming, another parameter, deformation velocity, is introduced here which is defined by:

(6) $$\mathrm{Deformation}\;\mathrm{velocity}=\frac{\mathrm{Deformation}\;\mathrm{coefficient}-1}{\mathrm{pulse}\;\mathrm{duration}}\;$$

The comparation of maximum DC and deformation velocity under these two excitations is given in Figure 5c. Resulted by the longer treating time in each cycle, cells under 15 s/15 s excitation show higher deformation extent. While the deformation velocity of 5 s/5 s excitation is significantly larger than that of 15 s/15 s excitation, which is due to the higher frequency of the stimulation used in 5 s/5 s excitation method.

These results demonstrated that this hydrodynamic force loading platform could realize controllable cell deformation following complex patterns. Benefited from the easy and flexible regulation of the electric signal and the instant conversion from the electric energy to the fluid motion, we can further assume that by applying a code‐controlled multiparameter signal with tunable power, pulse duration and duty cycle, a desired final cell shape or cell shape regulating trajectory can be automatically achieved, and a programmable cell behavior regulation platform can be extended.

### 2.4 Application of the Controllable Cell Stretching for Membrane Permeability Modulation

**Figure 6:**
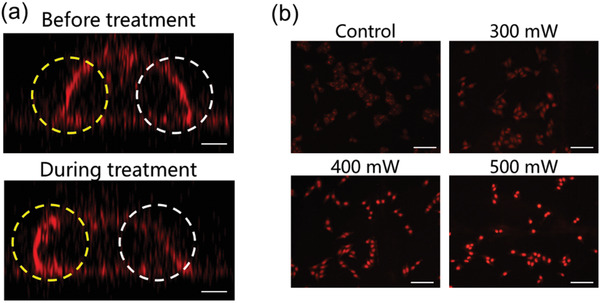
Cell membrane reconstruction and permeability change under streaming excitation. a) Cell membrane fluorescence before and during acoustic streaming treatment. White and yellow dashed circles indicate the pressed side and the back side of the cell, respectively. Scale bar is 5 µm. b) DOX uptake with or without acoustic streaming stimulation. Cells were treated under different power for 10 min. Scale bar is 100 µm.

We have observed an interesting phenomenon on our platform that during the cell deformation process, the membrane fluorescence intensity on one side of the cell which is facing the acoustic streaming tend to weaken, meanwhile the fluorescence on the other side tend to become stronger (**Figure** **6**a). Considering the fluidity of the plasma membrane, we assume that this variation in fluorescence is due to the change of membrane molecular density and construction. When the cell is pressed by the streaming, the flow along the cell surface will provide shear force to the membrane. This force may overcome the intermolecular forces in the membrane, compel the phospholipids to flow to the back side of the cell, and provide chances for crack formation on the membrane.

This unique feature indicates that the platform could be applied for membrane permeability study and manipulation. In order to confirm our assumption, penetration of DOX molecules through the cell membrane under streaming stimulation was evaluated to reveal the change in cell membrane permeability. To achieve larger area of treatment, distance between the cell substrate and the resonator was increased to 1 mm. As simulated in Figure S9 (Supporting Information), the vortex area is significantly increased under this condition. A relatively uniform force distribution on cells within 1 mm diameter area can be seen, thus enables simultaneous treatment on multiple cells. DOX fluorescence inside the Hela cells without and with streaming excitation under different power was observed, respectively. As shown in Figure 6b, low fluorescence intensity of the cells with no hydrodynamic force treatment indicates a low permeability of the membrane. The increased DOX fluorescence is observed after gigahertz stimulation, and it is power dependent. This result is consistent with the deformation result in Figure 4, where a larger power gives rise to a higher shape deformation efficiency. Thus, we can confirm that cell deformation can lead to crack formation on the cell membrane, and the permeability to molecules can be controlled by tuning the cell deformation extent. This permeability change was proved to be reversible, and the membrane permeability reversibility and cell viability under acoustic streaming excitation are given in Figures S10 and S11 in the Supporting Information. Besides, we found that cell aggregation status also influences cell deformation and membrane permeability, which is thoroughly discussed in Figures S12 and S13 in the Supporting Information.

**Figure 7:**
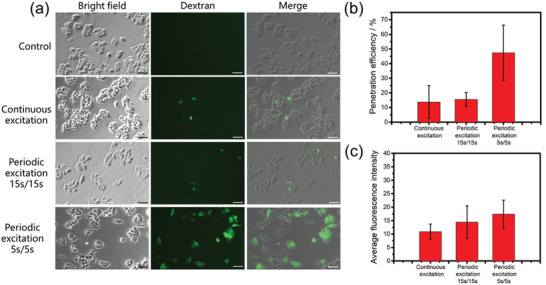
Membrane permeability for dextran under different excitations. a) Penetration of 40k FITC‐dextran molecules into HeLa cells using different excitation methods. Each group was treated in 2 mg mL^−1^ dextran solution under 500 mW power for 10 min. Scale bar equals to 50 µm. Quantitative analysis of the dextran penetration efficiency (indicating the percentage of the dextran‐delivered cells in view) and the mean fluorescence intensity in delivered cells are given in b,c) (*n* = 3).

We further performed the cellular uptake experiment with larger molecules (FITC‐dextran, *M*w 40K), as shown in **Figure** **7**. The control group and experiment group with 500 mW continuous stimulation shows obvious difference in the dextran penetration efficiency, which proves that the continuous cell deformation process can also alter the membrane permeability for larger biomolecules entering, however the penetration efficiency only reaches about 10%. In order to further optimize the membrane permeability for macromolecules, two cyclic hydrodynamic excitation methods which have been studied in Figure 5, 5 s/5 s and 15 s/15 s square‐waved excitations, were applied in this experiment. Compared with the continuous excitation, 15 s/15 s excitation shows no obvious improvement in intracellular fluorescence. However, under 5 s/5 s treatment, the dextran penetration efficiency is significantly increased, and the average fluorescent intensity in the dextran‐delivered cells is also much higher than that of continuous excitation.

The result indicates that fast and repeating periodic cell squeezing can induce remarkable effect on increasing the membrane permeability to macromolecules. A possible explanation for this phenomenon is that when the cell is treated with a stable force, the cell deformation and membrane phospholipid distribution will reach an equilibrium status. In contrast, the cell shape will be in a continuously changing state by alternatively apply and withdraw forces; the cell membrane experiences an extruding‐relaxing process, and there will be a mechanical accumulative effect, which facilitates the membrane molecules break their force balance and in favor of the macromolecule membrane transportation. Such cell membrane dynamic fatigue behaviors have been reported before for red blood cells treatment under cyclic stretching loads.^[^ ^45^, ^46^ ^]^ An increase in the membrane shear modulus of red blood cells was found after several tens of cycles of cell stretching and extensional recovery, indicating a decrease in elasticity and an increase in membrane brittleness in response to cyclic stresses. Besides, theoretical study which combines the cell resonance and cell fatigue theories was reported as well.^[^ ^47^ ^]^ They explain the effect of cell resonance using the cumulative effect under ultrasonically driven strains with a smaller magnitude but enormous number of loading cycles. Thus, we assume that the enhanced dextran delivery under periodic cell deformation is likely from a fatigue‐like behavior on cell membrane, which facilitates the macromolecule transportation.

In summary, this GHz resonator based hydrodynamic force loading and cell deformation platform can serve as a novel tool for cell membrane permeability modulation. Our results have revealed the change of membrane permeability under different force environments. Most prominently, we have observed a novel regulation approach based on this platform that cells tend to produce larger cracks for macromolecules transportation under the fast and periodic oscillation. Considering the significance of the membrane permeability, this discovery may help with the in‐depth mechanism study and provide a novel way for the optimization of the cellular drug uptake efficiency. The future steps will be focused on improving the current system for real‐time simultaneous observation of cell deformation and drug penetration to deepen our understanding on fluid–membrane and molecule–membrane interactions.

## 3 Conclusion

In this article, we developed a novel hydrodynamic cell deformation platform based on acoustic streaming generated from a microfabricated gigahertz resonator. The direct conversion from acoustic energy to localized fluid motion of the gigahertz device enables the noncontact and pure fluid force manipulation on this platform, which avoids the need of bubble or structure assistance for energy transformation in traditional technologies, improves the system flexibility and reduces the difficulty to manipulate at high accuracy. By integrating the deformation platform with confocal microscope, real‐time 3D observation of cell deformation is realized, making it possible for the analysis of complex cell morphological response under different stimulation patterns. Our DOX and dextran uptake experiments further investigated the effect of hydrodynamically controlled cell deformation on membrane molecule construction. The results revealed a promising application area of this platform for cell membrane permeability modulation, strongly proved the potential value of this platform in developing new cell manipulation and cell therapeutic methods.

## 4 Experimental Section

##### Device Fabrication

Gigahertz acoustic resonator was fabricated as described in previous publication.^[^ ^35^ ^]^ Briefly, Bragg mirror structure consisting of alternating layers of silicon dioxide (SiO_2_) and aluminum nitride (AlN) was deposited on Si wafer for acoustic wave reflection. 600 nm molybdenum (Mo), 1000 nm AlN, 60 nm chromium (Cr), and 300 nm gold (Au) were then deposited and patterned to form a sandwich structure for acoustic vibration. The size of the resonator working area is 0.01 mm^2^.

##### Cells Preparation

HeLa cells were grown in Dulbecco's modified Eagle medium (DMEM) supplemented with 10% fetal bovine serum and 1% penicillin–streptomycin. The cell line was maintained in T‐25 cell culture flasks in an incubator at 37 °C and 5% CO_2_ level. For laser confocal observation, cells were cultured on glass bottom culture dishes. The bottom of the culture dish was precoated with Fibronectin from human plasma (0.2 mg mL^−1^, Solarbio, China) for cell adhesion. Cell suspension (0.5 × 10^5^ cells mL^−1^, 2 mL) was loaded and cultured for 2 d before experiment. For DOX (doxorubicin hydrochloride) and dextran delivery, cells were cultured on glass slides with same conditions.

##### System Setup

The acoustic resonator was controlled by a sinusoidal signal (1.64 GHz), which was generated by a signal generator (Agilent, N5171B) and preamplified by a power amplifier (Mini‐Circuits, ZHL‐5W‐422+). The resonator was wire‐bonded to evaluation boards for signal transmission. For confocal experiments, the resonator was held by a position control platform and was placed facing the bottom of the culture dish. In drug delivery experiments, a PDMS chamber with a thickness of 1 mm was sealed on the resonator to form a drug container and fulfilled with DOX or dextran solution (100 µL). Glass slides cultured with Hela cells were then covered on the chamber and in contact with the drug solution.

##### Acoustic Streaming Characterization

The resonator vibration and fluid force distribution along the resonator surface were characterized by AFM (Dimension Icon, Bruker Corp., Bremen, Germany), which were performed following the procedures described in previous publication.^[^ ^48^ ^]^ Briefly, the gigahertz resonator was actuated by an amplitude‐modulated signal during AFM detection. The carrier frequency was equal to the resonance frequency, and the modulation frequency was equal to the second resonance frequency (≈110 kHz) of the AFM cantilever. Thus, the AFM tip follows the envelope of the resonator or fluid vibration, and its response at the modulation frequency is proportional to the detected vibration amplitude. Considering the AFM detection range (90 µm in length), resonator with a smaller working area (0.005 mm^2^) was used in this experiment. AFM tip was placed at the surface of the resonator when mapping the device vibration, and was lifted for 100 nm (tip offset = 100 nm) when testing the fluid force to make sure that the tip was not influence by the solid surface. The final force value (*F*) was calculated by multiplying the spring constant (*k*, 0.35 N m^−1^), *d* the sensitivity (*S*, 70 nm V^−1^) of the cantilever and the response of electric voltage (*V*, which was giver by the AFM software):

(7) F=k∗S∗V

Force transducer (Aurora Scientific, 405A) with a tip of 60 µm in diameter was used to characterize acoustic streaming under different power excitation, and the value was extracted and recorded with a Source Measure Unit (Keithley 2400, USA). Fluid velocity was recorded using a self‐established particle tracing system. The resonator was immersed in DI water filled with fluorescent microparticles (15 µm diameter, FluoSpheres, Life technologies, OR, USA). Green laser (532 nm, 100 mW, LD‐WL206, Changchun New Industries Optoelectronics Tech. Co., China) and a cylindrical lens were used to generate a plane fluorescent illumination which passes through the center of the resonator. Particle movement was recorded using a high‐speed camera (Phantom v7.2, Vision Research, NJ, USA).

##### Finite Element Simulation

Simulation of acoustic streaming field and cell deformation process was given using the fluid‐structure interaction model in COMSOL Multiphysics 5.5. Gigahertz vibration induced acoustic streaming is governed by a decaying body force generated from acoustic attenuation, which is calculated following Equations ((1), (2), (3), (4)). In 2D simulation, body force area was set to 100 µm (width) * 50 µm (height). Liquid material is set to water, and the velocity field is described by the incompressible Navier‐Stokes equation which is given in COMSOL software. Cell was modeled using a semicircular‐shaped linear elastic material with a diameter of 20 µm with its straight side fixed to the fluid field boundary, and the cell elastic properties were used as published in previous publications.^[^ ^49^ ^]^ In 3D simulation, body force was given in an area with a pentagon‐shaped bottom (side length 100 µm) and 50 µm height. Cell was represented with a semisphere of 20 µm in diameter. Other settings are the same as the 2D simulation.

##### Cell Deformation Recording and Analysis

Confocal microscope (Leica, SP8) was used for cell deformation recording. Before experiments, cells were washed with 1× PBS for three times and stained the membrane with wheat germ agglutinin (Alexa Fluor 647 conjugate, Life Technologies, USA) (10 µg mL^−1^) for 20 min. After that, cells were rinsed with 1× PBS for another three times, and fresh PBS solution was added to the culture dish for the experiment. Culture dish was then placed on the stage of the confocal microscope, and repeated acquisition of complete volumes (xyzt scan mode) was used at an excitation wavelength of 633 nm to trace the cell shape before, during and after acoustic streaming treatment. The device was held by a manually controlled three‐axis linear translational stage with a 10 µm position accuracy for position control and was inserted into the PBS solution. The relative position of the cell and the resonator is tuned with the following procedures. Position of the device was firstly confirmed under a low power objective lens (20×), and the pentagon area was moved to the center of the view by tuning *x* and *y* axis of the stage. Then, the *z* position of the device was lowered down carefully until the device surface and the cells on the substrate are all clear in the same focal plane. This position was defined to be *z* = 0 position for the device, which means the distance between the device and the cells is 0. After that, the device was again carefully lifted for 100 µm, and the microscope was switched to a high‐power lens (60×) for cell scanning. The objective stage of the microscope together with the petri dish was moved to select cells for scanning, and the selected cells were placed at the center of the view to make sure that the cell is in the resonator streaming area.

##### Intracellular Delivery Recording

Doxorubicin hydrochloride (DOX, Aladdin, China) (2 µg mL^−1^) and fluorescein isothiocyanate labeled dextran (FITC‐Dextran, *M*w 40 000, Sigma‐Aldrich, USA) (2 mg mL^−1^) were used to characterize the cell membrane permeability and drug delivery efficiency. For each experiment, cells on the glass slides were treated with acoustic streaming for 10 min, then were washed with 1× PBS for three times. Fluorescence microscope (Olympus BX53) with a CCD camera (Olympus DP73) was utilized to observe the intracellular fluorescence. The distance between the resonator and the cells was controlled to 1 mm by sealing a 1 mm thickness round PDMS chamber in between. The resonator was fixed at the center of the round PDMS area, and only the cells at the center of the substrate (indicating cells opposite to the resonator area) were selected for fluorescence observation.

##### Cell Viability Evaluation

Cell viability change under acoustic streaming stimulation was evaluated using a MTT assay. Cells were seeded into a 96‐well plate at a density of 1.0 × 10^4^ cells per well in 200 µL culture medium and were grown for 1 d. Thereafter, the cells were stimulated with or without gigahertz resonator for 10 min, followed by another 48 h incubation. After that, MTT solution (5 mg mL^−1^, 10 µL) was added into each well and the cells were incubated for 4 h. Then the media was completely removed and the cells were washed with 1× PBS for three times. 150 µL of dimethylsulfoxide (DMSO) was added to each well, and the formazan was dissolved for 10 min on an orbital shaker at a shaking speed of 300 rpm. The absorbance was measured with a microplate reader at the wavelength of 490 nm.

##### Statistical Analysis

The data are presented as mean ± SD in fluid force measurements (*n* = 3), cell shape analysis (*n* = 3), intracellular fluorescence analysis (*n* = 3), and cell viability analysis (*n* = 5). Particle tracking for fluid velocity analysis was processed with TrackMate plugin in ImageJ software and plotted with 3D scatter function in MATLAB (MathWorks, USA). Cell cross‐sections were obtained with Leica Application Suite X. Extraction of cell outlines and calculation of intracellular fluorescence intensity were made in ImageJ software.

## Conflict of Interest

The authors declare no conflict of interest.

1

W.
Li
, 
Z.
Yan
, 
J.
Ren
, 
X.
Qu
, Chem. Soc. Rev.
2018, 47, 8639.3028396210.1039/c8cs00053k2

M.
Merkel
, 
M. L.
Manning
, Semin. Cell Dev. Biol.
2017, 67, 161.2749633410.1016/j.semcdb.2016.07.029PMC52902853

H.
Chen
, 
J.
Cornwell
, 
H.
Zhang
, 
T.
Lim
, 
R.
Resurreccion
, 
T.
Port
, 
G.
Rosengarten
, 
R. E.
Nordon
, Lab Chip
2013, 13, 2999.2372794110.1039/c3lc50123j4

J. D.
Humphrey
, 
E. R.
Dufresne
, 
M. A.
Schwartz
, Nat. Rev. Mol. Cell Biol.
2014, 15, 802.2535550510.1038/nrm3896PMC45133635

W.
Jiang
, 
D.
Niu
, 
L.
Wei
, 
G.
Ye
, 
L.
Wang
, 
H.
Liu
, 
P.
Chen
, 
F.
Luo
, 
B.
Lu
, Carbon
2018, 139, 1048.6

K. H.
Vining
, 
D. J.
Mooney
, Nat. Rev. Mol. Cell Biol.
2017, 18, 728.2911530110.1038/nrm.2017.108PMC58035607

C.
Guilluy
, 
L. D.
Osborne
, 
L.
Van Landeghem
, 
L.
Sharek
, 
R.
Superfine
, 
R.
Garcia‐Mata
, 
K.
Burridge
, Nat. Cell Biol.
2014, 16, 376.2460926810.1038/ncb2927PMC40856958

M.
Werner
, 
S. B. G.
Blanquer
, 
S. P.
Haimi
, 
G.
Korus
, 
J. W. C.
Dunlop
, 
G. N.
Duda
, 
D. W.
Grijpma
, 
A.
Petersen
, Adv. Sci.
2017, 4, 1600347.10.1002/advs.201600347PMC5323878282510549

N.
Huebsch
, 
E.
Lippens
, 
K.
Lee
, 
M.
Mehta
, 
S. T.
Koshy
, 
M. C.
Darnell
, 
R. M.
Desai
, 
C. M.
Madl
, 
M.
Xu
, 
X.
Zhao
, 
O.
Chaudhuri
, 
C.
Verbeke
, 
W. S.
Kim
, 
K.
Alim
, 
A.
Mammoto
, 
D. E.
Ingber
, 
G. N.
Duda
, 
D. J.
Mooney
, Nat. Mater.
2015, 14, 1269.2636684810.1038/nmat4407PMC465468310

A.
Sharei
, 
J.
Zoldan
, 
A.
Adamo
, 
W. Y.
Sim
, 
N.
Cho
, 
E.
Jackson
, 
S.
Mao
, 
S.
Schneider
, 
M. ‐ J.
Han
, 
A.
Lytton‐Jean
, 
P. A.
Basto
, 
S.
Jhunjhunwala
, 
J.
Lee
, 
D. A.
Heller
, 
J. W.
Kang
, 
G. C.
Hartoularos
, 
K. ‐ S.
Kim
, 
D. G.
Anderson
, 
R.
Langer
, 
K. F.
Jensen
, Proc. Natl. Acad. Sci. USA
2013, 110, 2082.2334163110.1073/pnas.1218705110PMC356837611

M.
Sun
, 
X.
Duan
, Nanotechnol. Precis. Eng.
2020, 3, 18.10.1016/j.npe.2019.12.001PMC80065653378642412

J.
Li
, 
B.u
Wang
, 
B. M.
Juba
, 
M.
Vazquez
, 
S. W.
Kortum
, 
B. S.
Pierce
, 
M.
Pacheco
, 
L.
Roberts
, 
J. W.
Strohbach
, 
L. H.
Jones
, 
E.
Hett
, 
A.
Thorarensen
, 
J. ‐. B.
Telliez
, 
A.
Sharei
, 
M.
Bunnage
, 
J. B.
Gilbert
, ACS Chem. Biol.
2017, 12, 2970.2908852810.1021/acschembio.7b0068313

X.
Han
, 
Z.
Liu
, 
M. C.
Jo
, 
K.
Zhang
, 
Y.
Li
, 
Z.
Zeng
, 
N.
Li
, 
Y.
Zu
, 
L.
Qin
, Sci. Adv.
2015, 1, e1500454.2660123810.1126/sciadv.1500454PMC464379914

A.
Sawicka
, 
A.
Babataheri
, 
S.
Dogniaux
, 
A. I.
Barakat
, 
D.
Gonzalez‐Rodriguez
, 
C.
Hivroz
, 
J.
Husson
, Mol. Biol. Cell
2017, 28, 3229.2893160010.1091/mbc.E17-06-0385PMC568702515

E. A.
Evans
, 
R. M.
Hochmuth
, Biophys. J.
1976, 16, 13.124488710.1016/S0006-3495(76)85659-7PMC133481016

M.
Lekka
, 
D.
Gil
, 
K.
Pogoda
, 
J.
Dulińska‐Litewka
, 
R.
Jach
, 
J.
Gostek
, 
O.
Klymenko
, 
S.
Prauzner‐Bechcicki
, 
Z.
Stachura
, 
J.
Wiltowska‐Zuber
, 
K.
Okoń
, 
P.
Laidler
, Arch. Biochem. Biophys.
2012, 518, 151.2220975310.1016/j.abb.2011.12.01317

Q.
Guo
, 
S. P.
Duffy
, 
K.
Matthews
, 
A. T.
Santoso
, 
M. D.
Scott
, 
H.
Ma
, J. Biomech.
2014, 47, 1767.2476787110.1016/j.jbiomech.2014.03.03818

J. R.
Lange
, 
C.
Metzner
, 
S.
Richter
, 
W.
Schneider
, 
M.
Spermann
, 
T.
Kolb
, 
G.
Whyte
, 
B.
Fabry
, Biophys. J.
2017, 112, 1472.2840288910.1016/j.bpj.2017.02.018PMC538996219

Y.
Zhang
, 
F.
Wei
, 
Y.‐C.
Poh
, 
Q.
Jia
, 
J.
Chen
, 
J.
Chen
, 
J.
Luo
, 
W.
Yao
, 
W.
Zhou
, 
W.
Huang
, 
F.
Yang
, 
Y.
Zhang
, 
N.
Wang
, Nat. Protoc.
2017, 12, 1437.2868658310.1038/nprot.2017.042PMC555516920

S.
Hu
, 
L.
Eberhard
, 
J.
Chen
, 
J. C.
Love
, 
J. P.
Butler
, 
J. J.
Fredberg
, 
G. M.
Whitesides
, 
N.
Wang
, Am. J. Physiol.: Cell Physiol.
2004, 287, C1184.1521305810.1152/ajpcell.00224.200421

X.
Liu
, 
R.
Liu
, 
Y.
Gu
, 
J.
Ding
, ACS Appl. Mater. Interfaces
2017, 9, 18521.2851414210.1021/acsami.7b0402722

A.
Sutton
, 
T.
Shirman
, 
J. V. I.
Timonen
, 
G. T.
England
, 
P.
Kim
, 
M.
Kolle
, 
T.
Ferrante
, 
L. D.
Zarzar
, 
E.
Strong
, 
J.
Aizenberg
, Nat. Commun.
2017, 8, 14700.2828711610.1038/ncomms14700PMC535580923

C.‐W.
Lai
, 
S.‐K.
Hsiung
, 
C.‐L.
Yeh
, 
A.
Chiou
, 
G.‐B.
Lee
, Sens. Actuators, B
2008, 135, 388.24

J.
Czerwinska
, 
S. M.
Wolf
, 
H.
Mohammadi
, 
S.
Jeney
, Cell. Mol. Bioeng.
2015, 8, 258.25

J. Y.
Hwang
, 
J.
Kim
, 
J. M.
Park
, 
C.
Lee
, 
H.
Jung
, 
J.
Lee
, 
K. K.
Shung
, Sci. Rep.
2016, 6, 27238.2727336510.1038/srep27238PMC489770726

L. A.
Macqueen
, 
M. D.
Buschmann
, 
M. R.
Wertheimer
, J. Micromech. Microeng.
2010, 20, 065007.27

I.
Doh
, 
W. C.
Lee
, 
Y. ‐ H.
Cho
, 
A. P.
Pisano
, 
F. A.
Kuypers
, Appl. Phys. Lett.
2012, 100, 173702.2258635510.1063/1.4704923PMC335053428

Y.
Xie
, 
N.
Nama
, 
P.
Li
, 
Z.
Mao
, 
P.o‐H.
Huang
, 
C.
Zhao
, 
F.
Costanzo
, 
T. J.
Huang
, Small
2016, 12, 902.2671521110.1002/smll.201502220PMC487696529

F.
Yuan
, 
C.
Yang
, 
P.
Zhong
, Proc. Natl. Acad. Sci. USA
2015, 112, E7039.2666391310.1073/pnas.1518679112PMC469742930

A. M.
Forsyth
, 
J.
Wan
, 
W. D.
Ristenpart
, 
H. A.
Stone
, Microvasc. Res.
2010, 80, 37.2030399310.1016/j.mvr.2010.03.00831

D. R.
Gossett
, 
H. T. K.
Tse
, 
S. A.
Lee
, 
Y.
Ying
, 
A. G.
Lindgren
, 
O. O.
Yang
, 
J.
Rao
, 
A. T.
Clark
, 
D.
Di Carlo
, Proc. Natl. Acad. Sci. USA
2012, 109, 7630.2254779510.1073/pnas.1200107109PMC335663932

S. S.
Lee
, 
Y.
Yim
, 
K. H.
Ahn
, 
S. J.
Lee
, Biomed. Microdevices
2009, 11, 1021.1943449810.1007/s10544-009-9319-333

A.
Sohrabi Kashani
, 
M.
Packirisamy
, AIMS Biophys.
2017, 4, 400.34

H.
Qu
, 
Y.
Yang
, 
Y.e
Chang
, 
Z.
Tang
, 
W.
Pang
, 
Y.
Wang
, 
H.
Zhang
, 
X.
Duan
, Sens. Actuators, B
2017, 248, 280.35

X.
Guo
, 
H.
Zhang
, 
Y.
Wang
, 
W.
Pang
, 
X.
Duan
, J. Nanobiotechnol.
2019, 17, 86.10.1186/s12951-019-0518-7PMC66834363138758136

S.
Pan
, 
H.
Zhang
, 
W.
Liu
, 
Y.
Wang
, 
W.
Pang
, 
X.
Duan
, ACS Sens.
2017, 2, 1175.2873081510.1021/acssensors.7b0029837

W.
Cui
, 
M.
He
, 
Y.
Yang
, 
H.
Zhang
, 
W.
Pang
, 
X.
Duan
, Part. Part. Syst. Charact.
2018, 35, 1800068.38

Z.
Zhang
, 
Y.
Wang
, 
H.
Zhang
, 
Z.
Tang
, 
W.
Liu
, 
Y.
Lu
, 
Z.
Wang
, 
H.
Yang
, 
W.
Pang
, 
H.
Zhang
, 
D.
Zhang
, 
X.
Duan
, Small
2017, 13, 1602962.39

Y.
Lu
, 
W. C.
De Vries
, 
N. J.
Overeem
, 
X.
Duan
, 
H.
Zhang
, 
H.
Zhang
, 
W.
Pang
, 
B. J.
Ravoo
, 
J.
Huskens
, Angew. Chem., Int. Ed. Engl.
2019, 58, 159.3041751810.1002/anie.201810181PMC639193840

M.
Ovchinnikov
, 
J.
Zhou
, 
S.
Yalamanchili
, J. Acoust. Soc. Am.
2014, 136, 22.2499319210.1121/1.488191941

N.
Li
, 
J.
Hu
, 
H.
Li
, 
S.
Bhuyan
, 
Y.
Zhou
, Appl. Phys. Lett.
2012, 101, 093113.42

P.o‐H.
Huang
, 
Y.
Xie
, 
D.
Ahmed
, 
J.
Rufo
, 
N.
Nama
, 
Y.
Chen
, 
C. Y.u
Chan
, 
T. J.
Huang
, Lab Chip
2013, 13, 3847.2389679710.1039/c3lc50568ePMC398890743

W.
Pang
, 
H.
Zhao
, 
E. S.
Kim
, 
H.
Zhang
, 
H.
Yu
, 
X.
Hu
, Lab Chip
2012, 12, 29.2204525210.1039/c1lc20492k44

W.
Cui
, 
W.
Pang
, 
Y.
Yang
, 
T.
Li
, 
X.
Duan
, Nanotechnol. Precis. Eng.
2019, 2, 15.45

Y.
Qiang
, 
J.
Liu
, 
E.
Du
, Acta Biomater.
2017, 57, 352.2852662710.1016/j.actbio.2017.05.03746

S.
Sakuma
, 
K.
Kuroda
, 
C.‐.H. D.
Tsai
, 
W.
Fukui
, 
F.
Arai
, 
M.
Kaneko
, Lab Chip
2014, 14, 1135.2446384210.1039/c3lc51003d47

M.
Or
, 
E.
Kimmel
, Ultrasound Med. Biol.
2009, 35, 1015.1937663810.1016/j.ultrasmedbio.2008.11.01148

F.
Xu
, 
X.
Guo
, 
L.
Xu
, 
X.
Duan
, 
H.
Zhang
, 
W.
Pang
, 
X.
Fu
, Micromachines
2017, 8, 244.10.3390/mi8080244PMC61899513040043549

W.
Kim
, 
A.
Han
, in The 14th Int. Conf. on Miniaturized Systems for Chemistry and Life Sciences (MicroTAS 2010), (Eds: 
S.
Verpoorte
, 
H.
Andersson‐Svahn
, 
J.
Emnéus
, 
N.
Pamme
) Vol. 1, Chemical and Biological Microsystems Society (CBMS), San Diego, California
2010, 253.

## Supporting information

Supporting Information

Supplemental Video 1

Supplemental Video 2

Supplemental Video 3

Supplemental Video 4

Supplemental Video 5

Supplemental Video 6

Supplemental Video 7
