# Supplementary material for: Controllable Cell Deformation Using Acoustic Streaming for Membrane Permeability Modulation
Source: Adv Sci (Weinh). 2020 Dec 21;8(3):2002489. doi: 10.1002/advs.202002489 (PMC7856903; doi:10.1002/advs.202002489)
Supplement: Supplementary file 1 — Supporting Information [file ADVS-8-2002489-s001.pdf]

© 2020 Wiley-VCH GmbH

ADVANCED SCIENCE Open Access

Supporting Information

for *Adv. Sci.,* DOI: 10.1002/advs.202002489

Controllable Cell Deformation Using Acoustic Streaming for Membrane Permeability Modulation

*Xinyi Guo#, Mengjie Sun#, Yang Yang, Huihui Xu, Ji Liu, Shan He, Yanyan Wang, Linyan Xu, Wei Pang, and Xuexin Duan**

Supplementary Figures

S1. Acoustic streaming field under different resonator size

Both frequency and the size of the resonator influences the acoustic streaming field. As we discussed in the manuscript, the frequency of the acoustic wave influences the body force, which further controls the speed of the acoustic streaming. The size of the resonator, as shown in the simulation in Figure S1, influences both the spatial range and the velocity distribution. A smaller resonator size will confine the body force in a smaller area, thus generates a more focused streaming field.

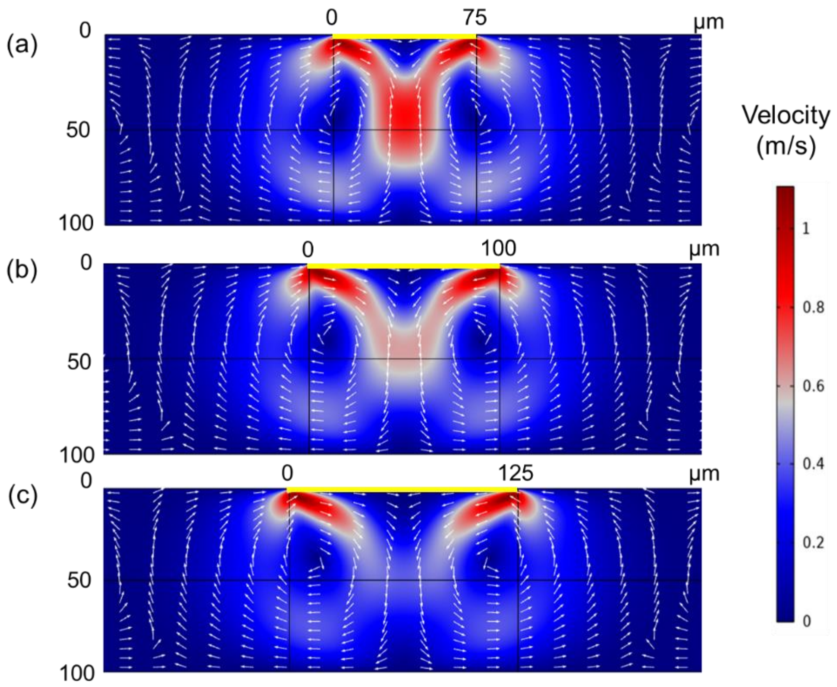

Figure S1 Acoustic streaming field generated by different sized resonator. Yellow rectangle indicates resonator area. Color bar and arrows indicate streaming direction and velocity.

S2. Acoustic streaming velocity distribution

Acoustic streaming velocity distribution recorded by high speed camera was given in Figure S2, in which a maximum longitudinal velocity of over 0.5m/s under 300 mW input power near the resonator can be seen.

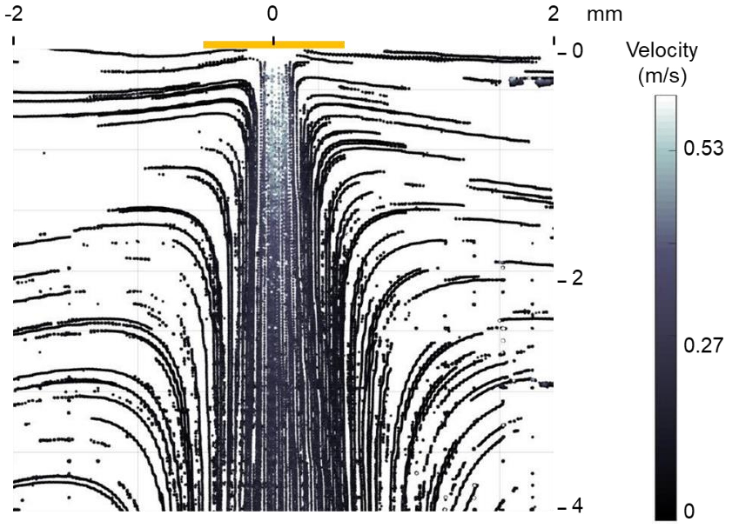

Figure S2 Streaming field recorded by high speed camera under 300 mW excitation. Yellow rectangle indicates the resonator position. Color bar indicate the vertical velocity of the trapped particles at different positions.

S3. Simulation of cell deformation at different horizontal positions.

To explore the influence of the horizontal relative position between the cell and the resonator on cell deformation, we did simulations at 4 different horizontal positions, and the results are given in Figure S3. When the cell is located directly opposite to the resonator center (position a), cell experiences a vertical impact force from the jet flow, and the cell is pressed from the top resulting a symmetric deformation. However, this phenomenon only appears when the cell is exactly at the central position, which is hard to realize in practical operation. When it's shifted to the side, the forces on two sides of the cell become unbalanced, and the deformation becomes oblique. When the cell is located at the edge of the resonator (position b), the lateral fluid flow extrudes the cell intensely. By moving the cell model further away from the resonator, as shown by position c and d, the deformation extent sharply decreased to almost zero. This is because the cell is removed from the main acoustic streaming area.

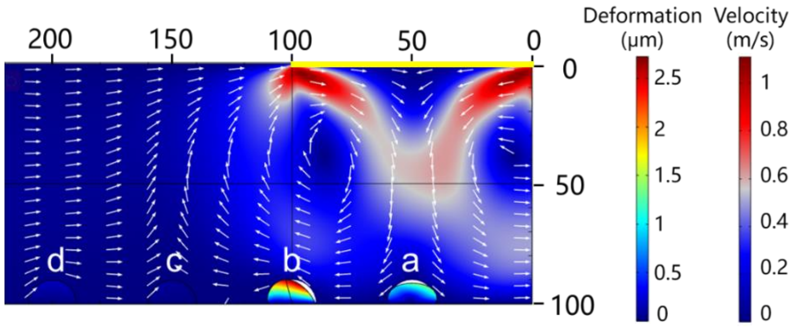

Figure S3. Simulation of the cell deformation at 4 different horizontal positions: opposite to the device center (position a); opposite to the device edge, which is 50 μm away from the center (position b); 100 μm (position c) and 150 μm (position d) away from the device center. Yellow rectangle indicates the position of the resonator. Due to the symmetry of the flow field, only half of the field is simulated. White arrows indicate flow directions, and color bars indicate streaming velocity distribution and cell deformation extent.

S4. 3D Simulation of cell deformation.

Figure S4 gives a 3D simulation of cell deformation, in which the relative vertical and horizontal distance between the cell and the resonator was set to the same condition as Figure 1(g). Comparing the results of 2D and 3D simulations, similar cell deformation feature and deformation extent can be seen.

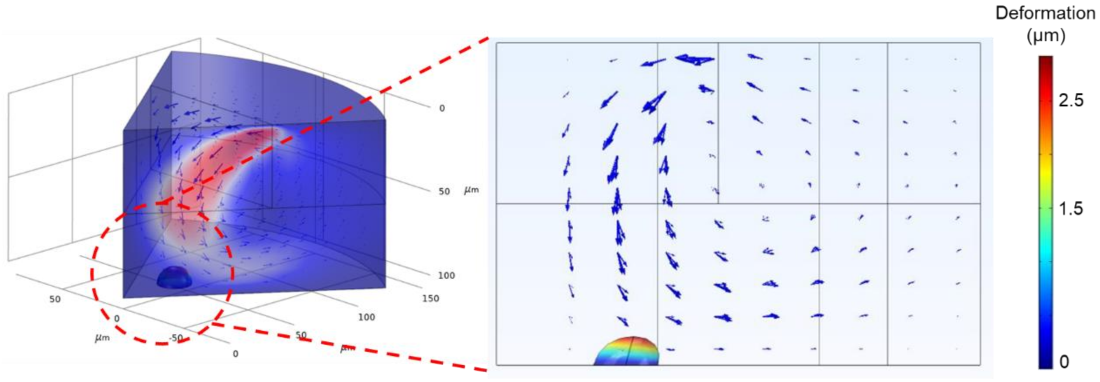

Figure S4 3D simulation result of cell deformation under 100 μm height.

S5. 3D cell recording by confocal microscope

Confocal microscope was used in our study for cell shape recording. Cell membrane was stained and excited under 633nm, and complete cell volumes were repeatedly recorded. Figure S5 shows the 3D recording of a static cell cluster.

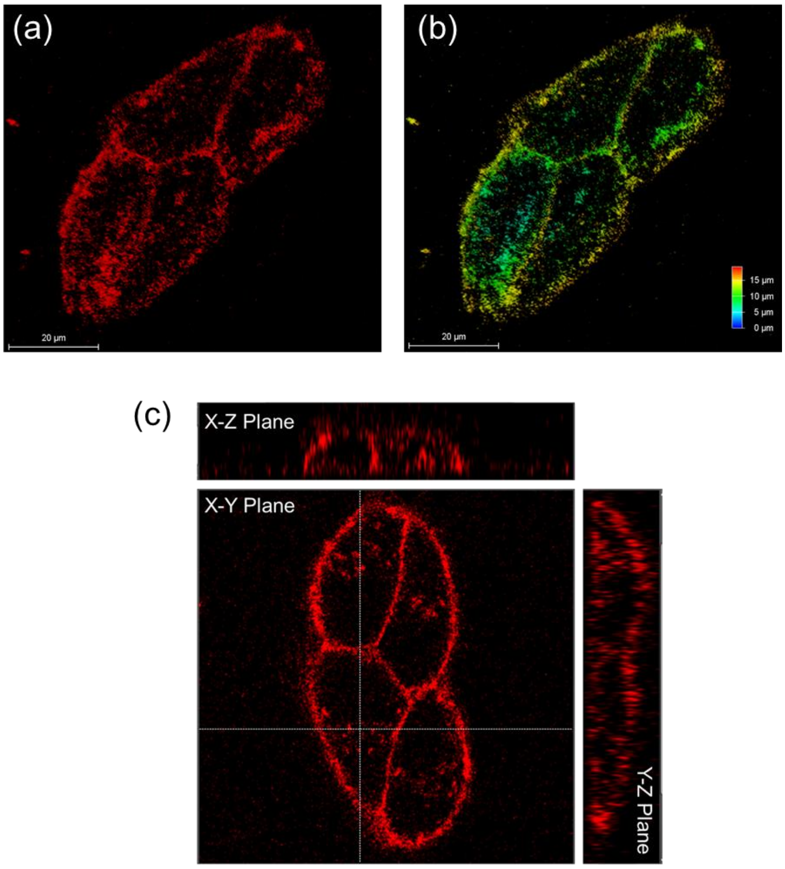

Figure S5. (a) (b) 3D view of a static cell cluster recorded by confocal microscope. The cells were displayed in its original fluorescent color (a) and in artificial color gradients for depth coding in Z direction (b). Orthogonal Slice views in three directions of the same cell cluster is given in (c).

S6. Comparing the deformation between a single cell and a cell cluster

In our experiments, cells were cultured for 2 days before observation to guarantee that the cells are fully attached and extended on the substrate, and normal well dispersed cells with good proliferative function should have already grown into a cell cluster. Thus, to assure that the tested cells are under good condition, experiments in Figure 3 to 7 were all performed using a cell in a small cell cluster. In order to discuss whether the cell aggregation status will also influence cell deformation, we also performed our experiments on a single cell with no connection and support from other adjacent cells in the petri dish, as shown in Figure S6. The cell comes from the same source as other experiments and was cultured and treated under the same condition. 300mW power was applied to the cell, and its deformation process is extracted. We can see from the pictures that there's almost no cell shape variation after 10s; on the other hand, the cell position is continuously moving along the force direction during the whole stimulation period. After turning off the power, cell shape gradually restored, but an unrecoverable cell position offset of about 5.5 μm appears. It's more obvious from Supplementary Video S6 that the cell is actually rolling on the surface along the deformation direction, which is a specific phenomenon that cannot be seen in cell clusters. We think these differences come from the different cell condition and also cell-cell interaction. Connections between cells provide supports for each other, thus confine cell deformation and displacement. Besides, a weaker cell adhesion force between the cell and the substrate may also lead to this phenomenon. In summary, the experiment also reveals the possibility of the platform to be applied for the cell status filtration.

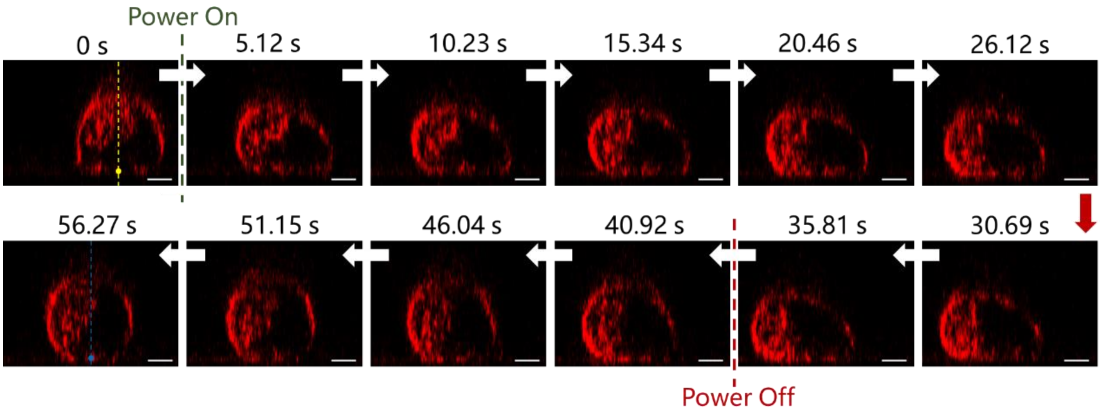

Figure S6. Deformation of a single cell under 300mW power treatment. Yellow dot and dash line indicate the original center position of the single cell before streaming treatment, and the blue ones indicate its final position after treatment. Scale bar is 5 μm. Besides cell shape variation, an obvious cell position shift after the stimulation was withdrawn can be seen.

S7. Cell detachment study under acoustic streaming stimulation

Cell detachment under our experimental condition was estimated based on previous living cell peeling models [1-4]. E. Décavé et al. [2] proposed a detachment rate constant, $k(\sigma)$, which is governed by cell and substrate conditions, shear stress and treating time, to describe the dynamic process of flow induced cell detachment:

$$k(\sigma) = k_0 * \frac{\exp(\sqrt{\frac{\sigma}{4\sigma_0}})}{(\sigma/4\sigma_0)^{1/4}}$$

Where σ is the applied shear stress; intrinsic detachment rate k_0 and stress σ_0 are two experimentally fitted parameters to scale the detachment rate for a given condition. $k(\sigma)$ (min $^{-1}$) is the inverse of the average time needed to detach a cell from the substrate under a given shear stress σ. Here we give a rough estimation using the experimental date provided in previous article [1] that the $k_0 = (0.6 \sim 1.6) * 10^{-4}$ (min $^{-1}$) and $\sigma_0 = (6 \sim 8) * 10^{-2}$ (Pa) were calculated from dictyostelium discoideum cells cultured on dimethyldichlorosilane glass substrate. Shear stress σ used in our experiment is estimated from simulation under two height conditions, 100 μm and 1 mm, as given in Figure S7.

As shown in Figure S7a, under 100 μm distance, a maximum shear of about 40 Pa is obtained, by which a $k(\sigma)$ ranging from 1.28 to 18 (min $^{-1}$) can be calculated. Since the maximum treating time used in our experiment is 40s which corresponds to $k = 1.5$ (min $^{-1}$), the maximum shear stress applied in our system already near the edge of the critical stress value for cell detachment. In our experimental observation, cell displacement and cell detachment were only observed on a separated single cell with a small attaching area (Figure S6), or under even larger power intensities (larger than 500 mW). The difference between calculation and experiment may come from different substrate modification (Fibronectin in our experiment) and cell aggregation status.

Under 1 mm distance, as given in Figure S7b, a maximum shear of about 3.3 Pa is obtained, by which a $k(\sigma)$ ranging from 0.0008 to 0.0034 (min $^{-1}$) can be calculated. The

number indicates that at least 294 min treatment is needed to peel a cell from the substrate under this condition, which is far beyond the time we need for membrane permeability regulation (10 min).

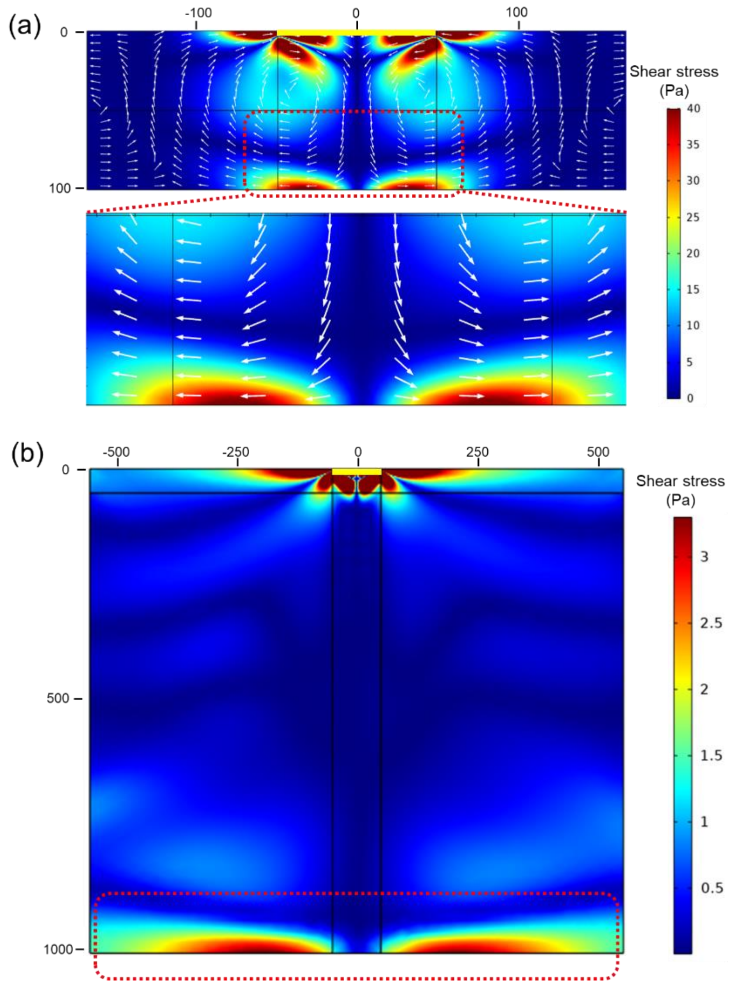

Figure S7 Shear stress distribution under a) 100 μm and b) 1 mm height. Yellow rectangle indicates resonator position and color bar indicates shear stress magnitude.

S8. Acoustic streaming characterization under two periodic excitation methods.

To figure out whether the acoustic streaming can be well controlled under pulsed and periodic gigahertz excitation, we real-time recorded the streaming force for 80s using 5s/5s and 15s/15s excitation methods using the force transducer under 500 mW power supply, as shown in Figure S8. The results shown that the generation of the acoustic streaming closely following the variation of the resonator vibration, indicating a rapid response and a good controllability of this force loading platform.

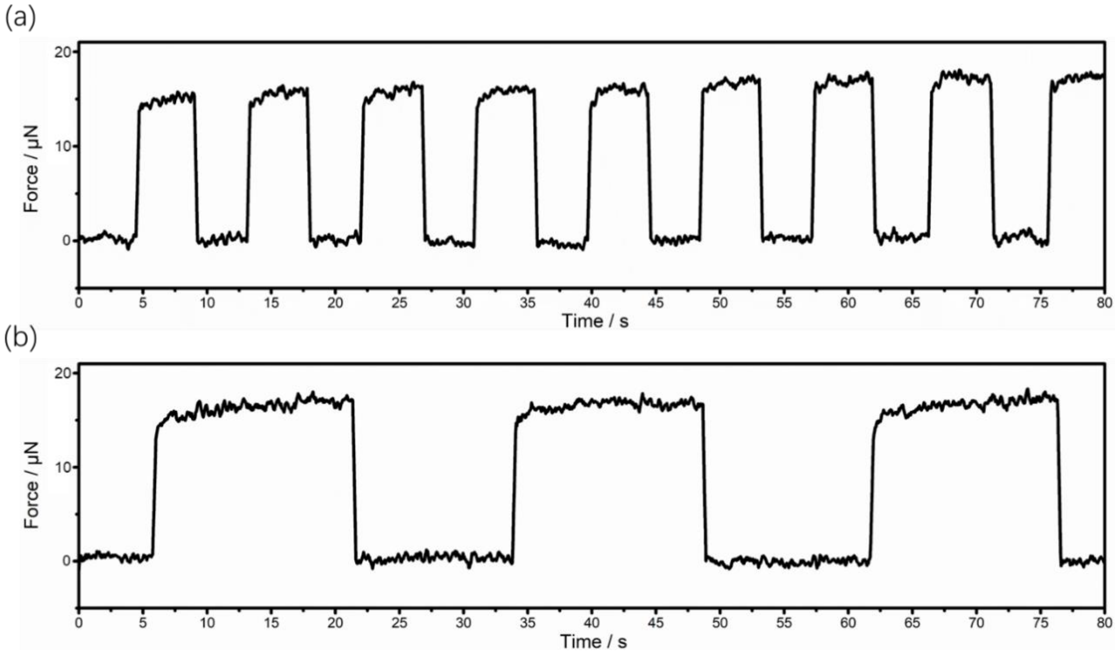

Figure S8. Acoustic streaming generated under 5s/5s (a) and 15s/15s (b) gigahertz excitation.

S9. Simulation of cell deformation at a height of 1mm.

In the cell deformation observation experiments, the distance between the resonator and the cells was controlled to 100 μm to obtain a distinct shape change. However, according to the simulation results, only cells near the resonator can be influenced due to the confined active range of the acoustic streaming under this height, which limits the cell batch processing in drug permeation experiments. In order to obtain a larger effective range, the distance between the cells and the resonator was set to 1 mm in membrane permeability tests. The simulation result shows that under this height, cells within about 1 mm diameter around the resonator can obtain a relatively uniform deformation.

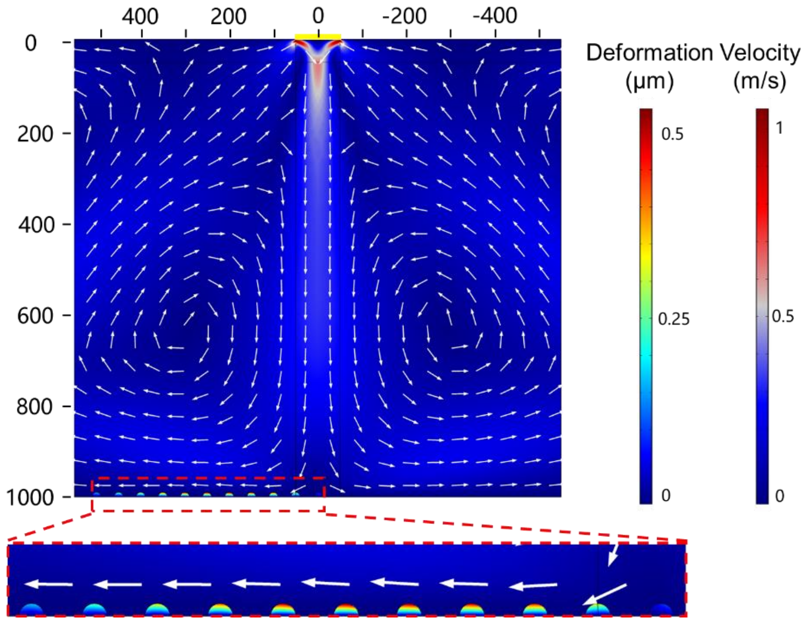

Figure S9. Simulation of cell deformation at a height of 1mm. The horizontal distance between each cell model is 50 μm. Yellow rectangle at the top center is the resonator. White arrows indicate flow directions, and color bars indicate streaming velocity distribution and cell deformation extent.

S10. Study on cell membrane permeability reversibility.

To prove that the cell membrane permeability is reversible, DOX delivery experiments during and after acoustic streaming excitation were carried out, as shown in Figure S10. Cells were firstly treated with acoustic streaming under 500 mW input power in 1X PBS solution for 10 min and then moved into the CO_2 incubator for another 10 min for cell membrane recovery. After that, as did in control group (Figure S10(a)), cells were immersed in 2 μg/ml DOX solution for 10 min (Figure S10(c)) to verify membrane permeability. Another group (Figure S10(b)) in which cells where treated with 500 mW acoustic power in 2 μg/ml DOX solution for 10 min was also conducted to serve as a positive control.

Figure S10 shows significant delivery efficiency in the positive control group, meanwhile no obvious difference in fluorescence intensity between the negative control and the membrane-recovered group was observed. The result indicates that membrane permeability change only occurs during the acoustic streaming treatment, and the cell membrane can recover to its original state in a rather short time period. Similar phenomenon was also reported in previous publications [5] that cell membrane rupture induced by micro-channel squeezing can recover within 5 min.

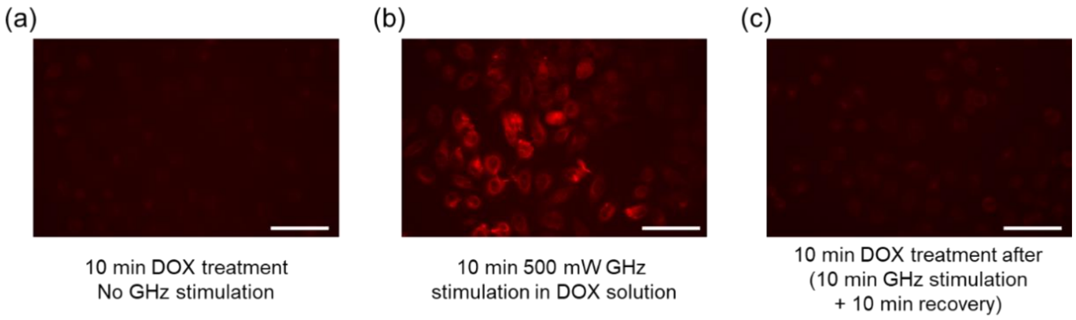

Figure S10 Study on cell membrane permeability reversibility. Intracellular DOX fluorescence was recorded under different excitation conditions. (a) Cells were immersed in DOX solution with no gigahertz device stimulation for 10 min. (b) Cells were treated under 500 mW power in DOX solution for 10 min. (c) Cells were firstly treated under 500 mW

power in 1X PBS for 10 min and then allowed to recover for 10 min followed by immersion in DOX solution for another 10 min. Scale bar is 100 µm.

S11. Cell viability change under acoustic streaming.

Cell viability change under acoustic streaming stimulation was evaluated using a MTT assay. The result is given in Figure S11. A slight cell viability decrease under 500 mW acoustic streaming stimulation was observed, and pulsed excitation shows less damage to cells.

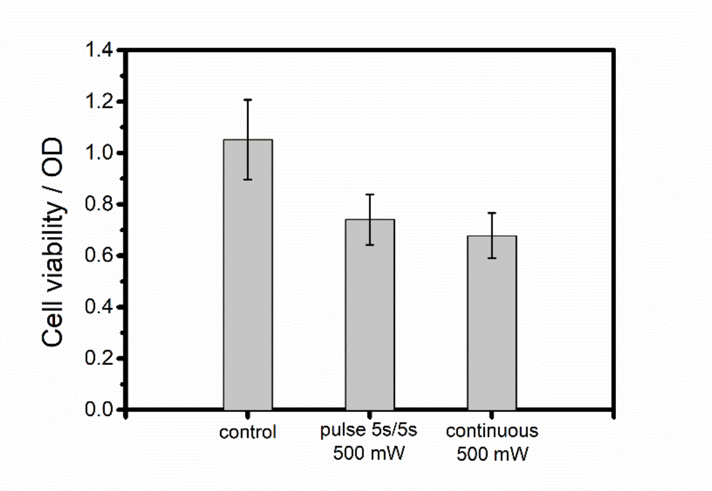

Figure S11 Cell viability change under different acoustic streaming treating conditions (n=3).

S12. Simulation of acoustic streaming and cell deformation under different cell aggregation status

In order to study the influence of the surrounding cells on acoustic streaming and cell deformation, simulations were further performed under different cell aggregation conditions, as given in Figure S12. The vertical distance between the cells and the resonator was set to 1 mm, same as experiments in Figure 7. A cell at a fixed position which is 150 μm away from the resonator center was studied, and four different surroundings were simulated: (a) a single cell model; (b) cell models were separated with 30 μm distance in between to simulate a loosely distributed cell condition; (c) 3 cell models were closely arranged with the target cell at the center to simulate a relatively small cell cluster; (d) 7 cell models were closely arranged with the target cell at the center to simulate a relatively large cell cluster. The simulation result indicates that both streaming patterns around the cells and the cell deformation extent vary with the change of the cell aggregation condition. Similar cell deformation extent between Figure S12(a) and (b) can be seen, which means that when cells are loosely seeded, cell deformation will not be influenced by other cells in the environment. This is because that spaces in between enable the fluid to flow through, and interactions between the cell and the fluid force occurs along the entire cell membrane surface. When a cell is in contact with other cells, as shown in Figure S12(c) and (d), fluid flow among cells is partially blocked. Only the upper surface of the cell can be influenced by the streaming, thus limits the cell deformation. Comparing Figure S12(c) and (d), a larger cell cluster will induce a much smaller cell deformation. In real cell environments, cells in a cell cluster can have even larger contact surface area, thus the interaction between cells and the fluid could be further weakened. This phenomenon was proved in cell experiments as well. As shown in Figure S13, when densely packed cells occur in a large area, weaker intracellular delivery were observed and only cells near the edge of the cell cluster can be well stimulated.

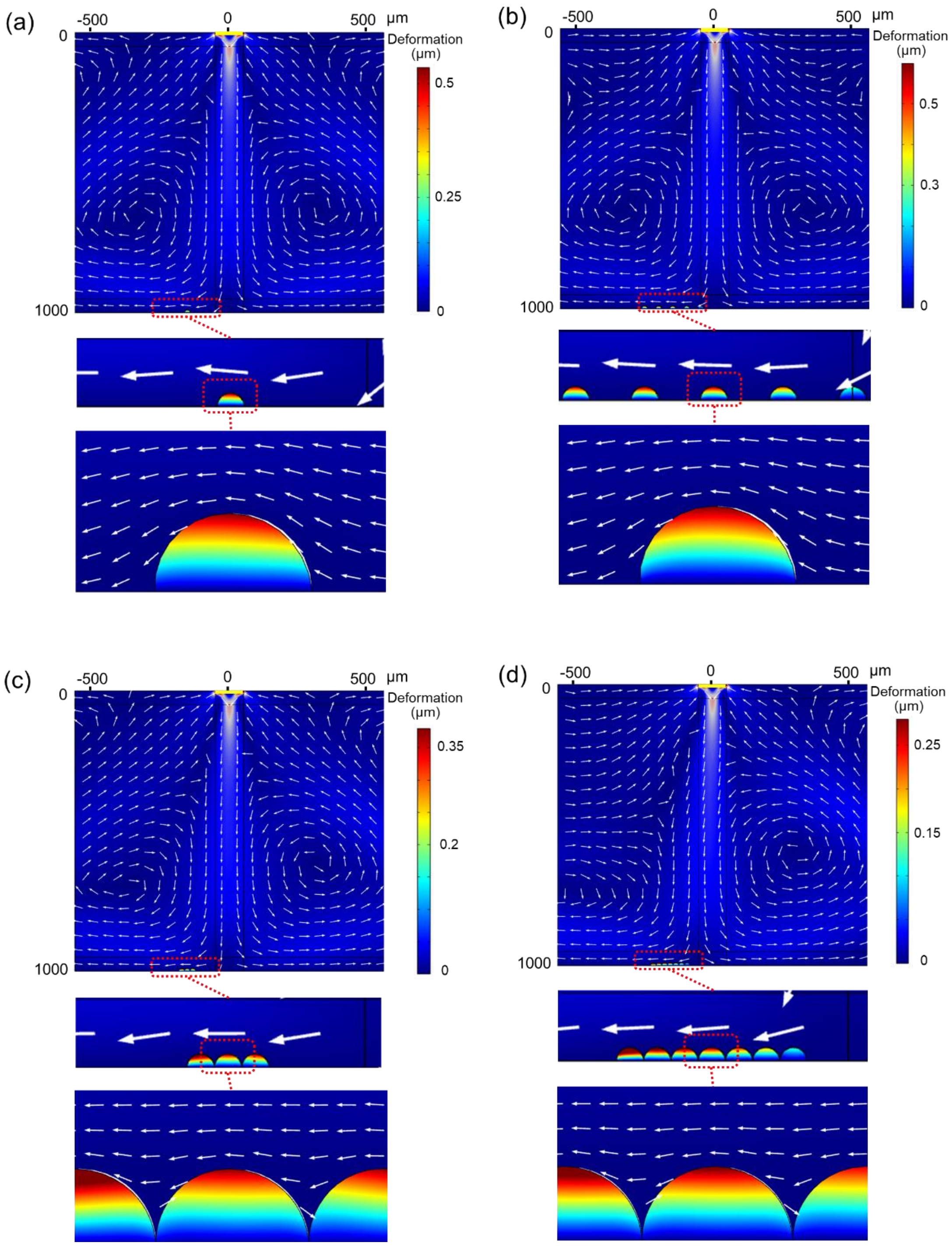

Figure S12 Finite element study under different cell aggregation status. A cell at 150 μm distance from the resonator center was selected for analysis under different surroundings: (a) a single cell; (b) cells were separated with 30 μm distance in between to simulate a loosely distributed cell condition; (c) 3 cells were closely arranged with the target cell at the center to simulate a relatively small cell cluster; (d) 7 cells were closely arranged with the target cell at

the center to simulate a relatively large cell cluster. Color bars indicate cell deformation extent, and arrows indicate flow directions.

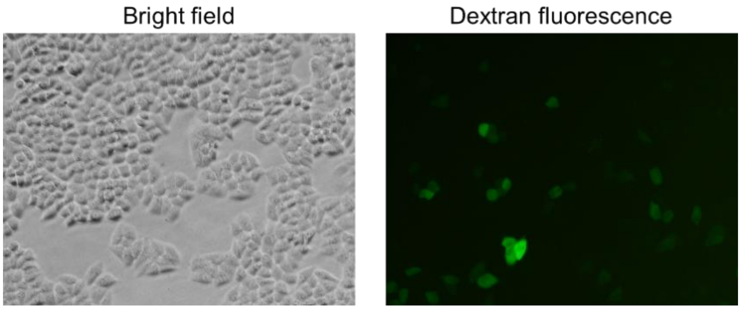

Figure S13 Intracellular dextran fluorescence under 500 mW 5s/5s pulse excitation when a high cell density is applied. The effect of acoustic streaming stimulation on cells in the over-dense region is weakened.

References

[1] E. Décavé, D. Garrivier, Y. Bréchet, B. Fourcade, F. Bruckert, *Biophys. J.* **2002,** *82,* 2383.

[2] D. Garrivier, E. Decave, Y. Brechet, F. Bruckert, B. Fourcade, *Eur. Phys. J. E: Soft Matter Biol. Phys.* **2002,** *8*, 79.

[3] Z. Tang, Y. Akiyama, K. Itoga, J. Kobayashi, M. Yamato, T. Okano, *Biomaterials* **2012,** *33*, 7405.

[4] R. Major, F. Bruckert, J. Lackner, W. Waldhauser, M. Pietrzyk, B. Major, *Bull. Pol. Acad. Sci.: Tech. Sci.* **2008**, 223.

[5] A. Sharei, R. Poceviciute, E. L. Jackson, N. Cho, S. Mao, G. C. Hartoularos, D. Y. Jang, S. Jhunjhunwala, A. Eyerman, T. Schoettle, R. Langer, K. F. Jensen, *Integr. Biol.* **2014,** *6*, 470.

Supplementary Videos

Supplementary Video S1: High-speed camera recorded acoustic streaming under 300 mW and 500 mW input power.

Supplementary Video S2: Simulation of the cell deformation process under acoustic streaming treatment.

Supplementary Video S3: Recorded 3D cell morphology (without streaming excitation) using Z-stacking function in confocal microscope. The 3D image was rotated to provide a dynamic view of the cell shape.

Supplementary Video S4: Cell deformation process under acoustic streaming. Shape change of the same cell cluster before, during and after the excitation was given in three ways: 3D inclined view, 3D top view and 2D sectional view.

Supplementary Video S5: Cell deformation under three different powers.

Supplementary Video S6: Comparation between the deformation of a single cell and a cell cluster.

Supplementary Video S7: Cell deformation under two kinds of periodic excitations, 5s/5s (indicating a square wave with 5s power on and 5s power off in a period of 10s) and 15s/15s (indicating a square wave with 15s power on and 15s power off in a period of 30s).
